# Performance of a self-developed panel for biogeographic ancestry inference and dissection of the genetic background of three Tibetan groups

**DOI:** 10.1186/s41065-025-00604-3

**Published:** 2025-12-05

**Authors:** Yifeng Lin, Xi Yuan, Xi Wang, Shuanglin Li, Hongbin Yao, Bonan Dong, Bofeng Zhu

**Affiliations:** 1 Guangzhou Key Laboratory of Forensic Multi-Omics for Precision Identification, School of Forensic Medicine, Southern Medical University, Guangzhou, Guangdong China; 2 Department of Anatomy and Histology, School of Basic Medical Sciences, Shenzhen University, Shenzhen, China; 3 Belt and Road Research Center for Forensic Molecular Anthropology, Key Laboratory of Evidence Science of Gansu Province, Gansu University of Political Science and Law, Lanzhou, Gansu China; 4 School of Forensic Medicine, Shanxi Medical University, Taiyuan, China

**Keywords:** Insertion/deletion polymorphism, Biogeographic ancestry inference, Machine learning, Tibetan group, Genetic structure

## Abstract

**Background:**

In this study, we used a panel consisting of 56 autosomal ancestry-informative insertion/deletions (AIM-InDels) for biogeographic ancestry inference, three Y-InDels, and one Amelogenin gene, and verified its performance in Gannan Tibetan, Qinghai Tibetan, and Tibet Tibetan groups. Meanwhile, we analyzed the genetic structures of these three Tibetan groups.

**Results:**

The results showed that 56 AIM-InDels performed better at classifying African and East Asian individuals without noisy labels compared with other intercontinental populations. By the addition of noisy labels, the SVM model was robust when the proportion of noisy labels was small. Furthermore, the African and East Asian populations showed better performance than the other three intercontinental populations. And the 56 AIM-InDels could be used for individual identification and full sibling identification of three Tibetan groups. Population genetic analysis of three Tibetan groups showed that their genetic structures were similar to East Asian populations.

**Conclusions:**

This panel can not only be effectively used for biogeographic ancestry inference in African and East Asian populations but also provide insights into the genetic structures of three Tibetan groups.

**Supplementary Information:**

The online version contains supplementary material available at 10.1186/s41065-025-00604-3.

## Background

Individual identification of biological sample at crime scene based on the short tandem repeat (STR) marker has been widely used in forensic casework [1]. When there are no other clues in the investigated case, if the STR genotyping result of the biological sample at the scene of the case is consistent with the result of existing sample in the forensic database, it will provide the valuable clue for us to identify the suspect. However, if there is a lack of information about the suspect in the forensic DNA database, we will be unable to locate the suspect using this method. In these cases, if we can use certain genetic markers to infer the biogeographic ancestry (BGA) of an unknown individual, this will help to narrow the scope of investigation to some extent [2]. The insertion and deletion (InDel) genetic marker refers to the insertion or deletion sequence of a small fragment of the human genome. Because of low mutation rate, short amplicon, no stutter peak, and compatibility with capillary electrophoresis platform, InDel can be used in forensic genetics [3]. In contrast, STR has a relatively high mutation rate, and it is difficult to obtain a complete DNA profile from degraded sample [4, 5]; single nucleotide polymorphism (SNP) genotyping suffers from time-consuming, costly, and complex experimental procedures [6]. Ancestry-informative InDels markers (AIM-InDels) can be used to infer BGA because their allele frequencies are significantly different among different populations. Previous research has extensively explored BGA inference based on the AIM-InDel panels. Liu et al. utilized 56 AIM-InDels and four machine learning models to evaluate the BGA efficacy of these AIM-InDels and to analyze the genetic structure of Chinese Inner Mongolia Manchu group [7]. Lan et al. used 41 multi-InDels for BGA inference and explored the genetic backgrounds of Manchu and Mongolian groups [8].

Machine learning technique have been widely applied in various fields, such as image recognition, speech recognition, medicinal chemistry, and so on [9–11]. In recent years, all kinds of machine learning methods have also been extensively utilized in the forensic field due to the rapid development of bioinformatics. Zolotenkova et al. used different machine learning models to assess biological age of the deceased based on postmortem osseous and cartilaginous tissues [12]. Tan-Torres et al. performed tissue origin identification based on the human microbiome using five different machine learning algorithms [13]. Previous studies have applied the machine learning to BGA inference. Sun et al. and Alladio et al. each constructed different BGA inference models using machine learning algorithms, respectively [14, 15]. Qu et al. proposed the use of a convolutional neural network for BGA inference [16]. However, classical machine learning models depond on high-quality datasets. Noisy data is randomly generated meaningless data that usually affects the analysis tasks of various data in machine learning [17]. The most common form of noisy data in classification problems is noisy labels. In the forensic BGA inference, it is manifested as the error of population label, which may be caused by the fact that the self-identified population of the sample donor during the sample collection process does not fully match its genetic background. If noisy labels are present in the dataset, the model’s performance will decrease. Support Vector Machine (SVM) is one of the most commonly used machine learning methods, which can solve high-dimensional problems and has a certain robustness [18]. Therefore, it is necessary to explore the effect of noisy labels in the dataset on the performance of SVM model.

According to the result of China’s seventh national census, the Tibetan group has a population of over 7 million (http://www.stats.gov.cn/tjsj/pcsj/rkpc/7rp/indexch.htm). Tibetans are mainly distributed in the Tibet Autonomous Region, Qinghai, western Sichuan, Yunnan, and Gansu provinces. The people living on the Qinghai-Tibet plateau have formed a plateau cultural system dominated by Tibetan culture. Previous research has mainly focused on Qinghai Tibetan (QHT) and Tibet Tibetan (TIT) groups and has found that the QHT and TIT groups are most similar in genetic structures to East Asian populations [19, 20]. However, they lacked research on other regions, such as the Tibetan group in Gannan Tibetan Autonomous Prefecture. Although studies on the Gannan Tibetan group (GNT) have been reported previously, they used SNP genetic markers and did not involve the research of the genetic relationships of the GNT group with QHT and TIT groups [21]. Therefore, it is necessary to explore the genetic backgrounds of Tibetan groups from different regions based on the AIM-InDel markers.

In this study, we used a panel consisting of 56 autosomal AIM-InDels for BGA inference, three Y-InDels, and one Amelogenin gene [22]. The SVM model was selected to evaluate the BGA inference performance of the panel mentioned above. The SVM model was established based on the 56 AIM-InDel data from 26 reference populations of the 1000 Genomes Project phase 3. Furthermore, we also explored the genetic structures of the GNT, QHT and TIT groups based on the self-developed panel.

## Methods

### Samples source and ethical declaration

The Southern Medical University and Xi’an Jiaotong University Ethics Committees reviewed and approved this research (No. 2019 − 1039). Before providing bloodstain samples, all the participants had already signed written informed consents. All 426 participants were healthy and unrelated, including 180 Tibetan individuals from Gannan Autonomous Prefecture of Gansu province, 144 Tibetan individuals from Qinghai province, and 102 Tibetan individuals from Tibet Autonomous Region. We selected these individuals who had no genetic relationships within at least three generations to ensure that they were unrelated to each other. The geographic locations of all populations are shown in the Figure. S1.

### Selection requirements of AIM- InDel loci

The AIM-InDel markers we selected from the 1000 Genomes Project database (http://grch37.ensem.bl.org) and the Nucleotide Polymorphism Database (https://www.ncbi.nlm.nih.gov/.

snp) followed the main criteria: (1) the minimal allele frequencies of AIM-InDel loci in the database were at least 0.1; (2) the fragment length of the insertion or deletion sequence was within 20 bp; (3) the allele frequency differences in selected loci were greater than 0.5 in the Asian, European, and African populations, and greater than 0.2 in the South American and American populations; (4) each AIM-InDel complied with Hardy-Weinberg equilibrium (HWE), and no linkage disequilibrium (LD) was observed in pairwise AIM-InDel loci. Other selection criteria can be referred to a previous study [23]. Detailed information about 60 selected loci is listed in Table S1.

### DNA extraction, PCR amplification, and capillary electrophoresis detection

The Chelex-100 method was utilized to isolate genomic DNA. The NanoDrop 2000 instrument measured the concentration of extracted genomic DNA (NanoDrop Technologies). DNA amplification was carried out using a GeneAmp^®^ PCR System 9700 Thermal Cycler (Thermo Fisher Scientific, Foster City, USA). The total volume of the amplification system was 10 µL, with 2 µL of 1.0× master mix, 2 µL of 1.0× primer, 2 µL of template DNA, and 4 µL of nuclease-free H_2_O. Thermal cycling conditions were set as follows: initial denaturation at 95 ℃ for 5 min; 2 cycles of 94 ℃ for 10 s and 63 ℃ for 90 s; 25 cycles of 94 ℃ for 10 s and 60 ℃ for 90 s; a final extension at 60 ℃ for 15 min; and kept at 4 ℃ for further analysis. 1 µL of PCR product was mixed with 8.5 µL of deionized formamide and 0.5 µL of internal standard (LIZ-500). And the mixture was further denaturized at 95 ℃ for 3 min and immediately incubated on ice for 2 min. Then the PCR products were detected on a 3500xL Genetic Analyzer (Thermo Fisher Scientific, Foster City, USA). The GeneMapper^®^ID-X v1.3 (Thermo Fisher Scientific, Foster City, USA) software was used to genotype all loci. We determined reliable allele calling for all loci by referencing a threshold of 50 relative fluorescence unit for peak heights. The positive control was DNA 9948, while the deionized water was used as the negative control.

### Data analysis

We used the PLINK program (version 1.9, https://www.cog-genomics.org/plink/1.9/) to download the ped file, and obtained 56 AIM-InDel data of a total of 2504 individuals from 26 populations from the 1000 Genomes Project phase 3 dataset [24] that we used as reference population data. Since sequence information is provided in the ped file, we changed the insertion sequence to allele 1 and the deletion sequence to allele 0 for each locus in the ped file, so as to be consistent with the data format of our Tibetan groups. Detailed information about reference populations is listed in Table S2.

The SVC function of the Python (version 3.10.7) SciKit-learn library [25] was used to evaluate the panel’s performance on the SVM algorithm for BGA inference. The kernel function of the SVM model was radial basis function (RBF), and the regularization parameter *C* and kernel coefficient gamma were adjusted. The dataset composed of the reference populations was divided into a training set and a testing set (80% and 20%, respectively). The Bayesian optimization method was used in the training set to adjust the hyperparameters (*C* and gamma) using 4-fold cross validation. The data from the three Tibetan groups was used as the external validation set, which was used to evaluate the model’s generalization ability. In addition, the labels in the reference populations were randomly shuffled to generate noisy labels to explore their influences on the model. In the training set, the proportions of normal data to noisy data were 99:1, 9:1, 4:1, 3:2, 1:1, 2:3, 3:7 and 2:8, respectively. The F1 score was calculated to assess the performance and generalization ability of the SVM model. Since a part of the data was randomly selected as the noisy data in the training set, the data set of each proportion was repeated 10 times to reduce this random effect, and the average value of the 10 times was used as the result.

Then, the forensic characteristics and genetic structures of the three Tibetan groups were dissected. We used the STRAF tool (version 1.0.5) [26] to calculate the allele frequencies, forensic parameters of 56 AIM-InDels in the three Tibetan groups, including the Ho, He, PIC, MP, PD and PE, and *P* values of HWE and pairwise LD tests. The forensic parameters of the three Tibetan groups and five intercontinental populations were visualized by the ‘ggplot2’ package [27] in *R* software (version 4.2.1). The ‘pheatmap’ package [28] was used to create a heatmap of insertion allele frequencies for 29 populations. Based on the allele frequencies of 56 AIM-InDels in three Tibetan groups, 1000 full sibling pairs and unrelated individuals, as well as 1000 half sibling pairs and unrelated individuals, were simulated using Familias v.3.0 software [29]. The H1 hypothesis is that the two individuals are full siblings or half siblings, while the H0 hypothesis is that the two individuals are unrelated. *F*-statistics (*F*_*ST*_) values and *Nei’s* genetic distances (*D*_*A*_ distances) of pairwise populations were calculated by the GenAlEx software (version 6.503) [30]. The heatmaps of *F*_*ST*_ values and *D*_*A*_ distances were plotted through the online website Chiplot (https://www.chiplot.online/). Informativeness for assignment (*I*_*n*_) values were calculated for the five intercontinental populations using the Infocalc program (version 1.1) [31] and visualized using the online website Chiplot.

The principal component analysis (PCA) was performed by the *R* packages ‘FactoMineR’, ‘factoextra’, and ‘corrplot ' [32]. Using the *R* packages ‘stats’ and ‘ggplot2’, a population-level multidimensional scaling (MDS) analysis based on pairwise *F*_*ST*_ values was performed. A phylogenetic tree was reconstructed based on pairwise *D*_*A*_ distances using the neighbor-joining (NJ) method by MEGA software (version 11.0.13) [33] and the online website Chiplot. In addition, Treemix software (version 1.13) [34] was used to infer the gene flow events of the 29 populations with its range from 0 to 11. Each gene flow event was iterated 10 times. The Evanno algorithm of the *R* package ‘OptM’ [35] was utilized to calculate the optimal number of gene flow events. The STRUCTURE program (version 2.3.4) [36] was used to analyze the ancestral components of 29 populations. The assumed ancestral component *K* values and number of iterations were set to 2 to 7, and 15, respectively. According to the InP*K* and delta*K* plots generated by the online website Structure Harvester [37], the optimal *K* value was measured. The average Q-matrices of the results of 15 iterations were calculated using CLUMPP software (version 1.1.2) [38]. Distruct software (version 1.1) [39], AncestryPainter program (version 1.1) [40] and the online website Chiplot were utilized to visualize 29 population ancestral components. The *R* package ‘ggtern’ [41] was employed to draw ternary plots based on the obtained ancestral components.

## Results

### HWE tests and LD analyses of 56 AIM-InDels in the three Tibetan groups

After applying Bonferroni correction (*P* > 0.05/56 = 0.00089), there were no significant deviations from the HWE tests at 56 AIM-InDels (Table S3). After applying Bonferroni correction (*P* > 0.05/1595 = 0.000031), no significant LDs were observed in the three Tibetan Tibetan groups (Tables S4-6).

### Performance of BGA inference for 56 AIM-InDels by SVM model

**Fig. 1:**
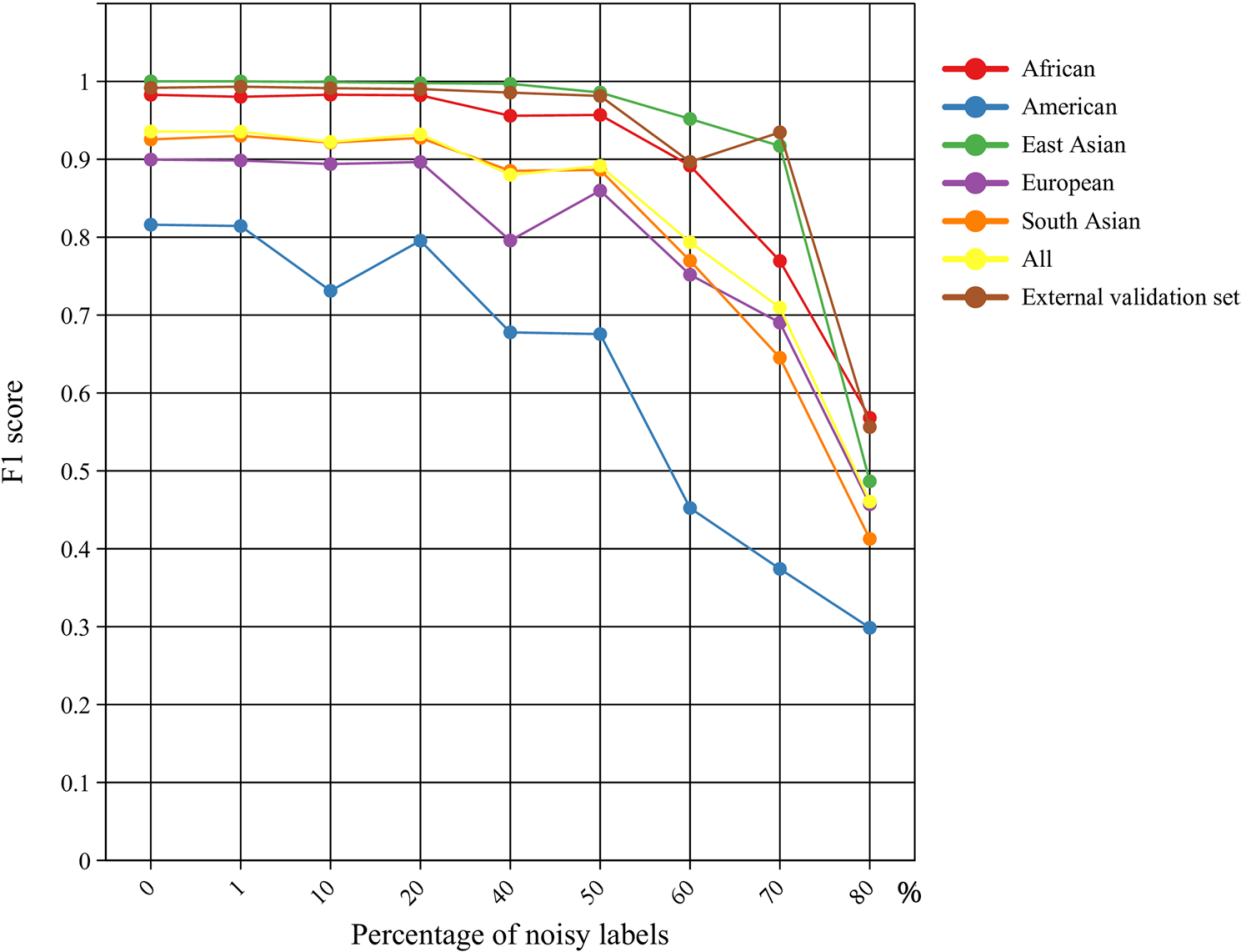
F1 scores for the testing and external validation sets under different percentages of noisy labels. As the percentage of noisy labels increased, the F1 scores of the testing set and external validation set showed decreasing trends. “All” in the legend meant that in the testing set, it was the F1 score of the five classification results

The SVM model was utilized to assess the BGA inference performance of 56 AIM-InDels. The F1 score is calculated based on precision and recall, while the weighted F1 score is calculated by considering the number of each category in the multi-classification case. In the absence of noisy data, the weighted F1 scores for the SVM model in the testing and external validation sets were 0.9357 and 0.9917, respectively. The classification results of five intercontinental populations were further investigated. As shown in Fig. 1, the 56 AIM-InDels have better classification results for African and East Asian individuals, but less effective for the American individuals. The 56 AIM-InDels could classify the individuals of three Tibetan groups into East Asian populations, demonstrating the panel’s ability to infer BGA in forensic practice.

When noisy data were introduced, Fig. 1 illustrated that the performance of the SVM model tended to decrease as the percentage of noisy labels increased. In the testing set, when the percentages of noisy labels were less than 50%, the performance of the SVM model remained well. For each intercontinental population, the classification performance of African and East Asian individuals remained strong, whereas European and South Asian individuals had a certain decline in classification performance. For the American population, the decline in classification performance was more obvious than that of other intercontinental populations. When the percentage of noisy labels reached 60%, the classification performance of the five intercontinental populations decreased significantly. In the external validation set, the trend of F1 score changes was basically the same as that of East Asian populations in the testing set. Among all five intercontinental populations, the panel performed the best for African and East Asian populations. The detailed F1 score values are shown in Table S6.

### Allele frequencies and forensic parameters of the 56 AIM-InDels in the three Tibetan groups

**Fig. 2:**
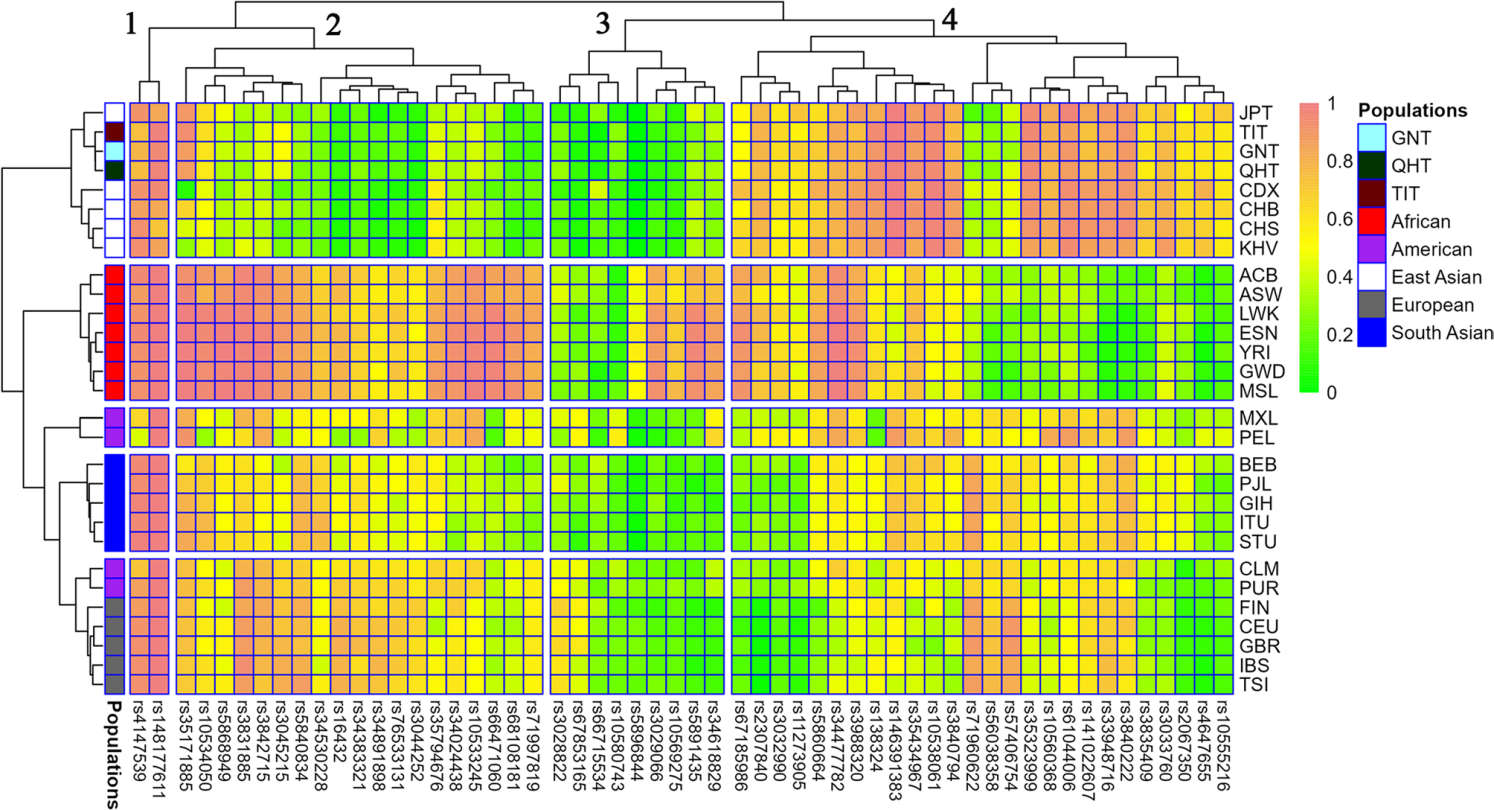
Heatmap of insertion allele frequencies in the three Tibetan groups and 26 reference populations. The color of the grid gradually changed from green to light coral representing their insertion allele frequencies from low to high. GNT: Gannan Tibetan; QHT: Qinghai Tibetan; TIT: Tibet Tibetan

After evaluating the performance of this panel for BGA inference, we used it to further characterize the genetic structures of the three Tibetan groups. Detailed allele frequencies of 56 AIM-InDels are listed in Table S3. Additionally, a heatmap based on their insertion allele frequencies of 56 AIM-InDels in 29 populations is generated (Fig. 2). The cluster analysis revealed that the 29 populations were divided into five clusters, with two American populations (CLM and PUR) clustered with European populations, and the three Tibetan groups located in the East Asian cluster. Cluster analysis of the 56 AIM-InDels classified them into clusters 1, 2, 3, and 4. We found that insertion allele frequency distributions at some loci varied in different intercontinental populations; for example, some AIM-InDels located in cluster 2 had higher insertion allele frequencies in African populations.

**Fig. 3:**
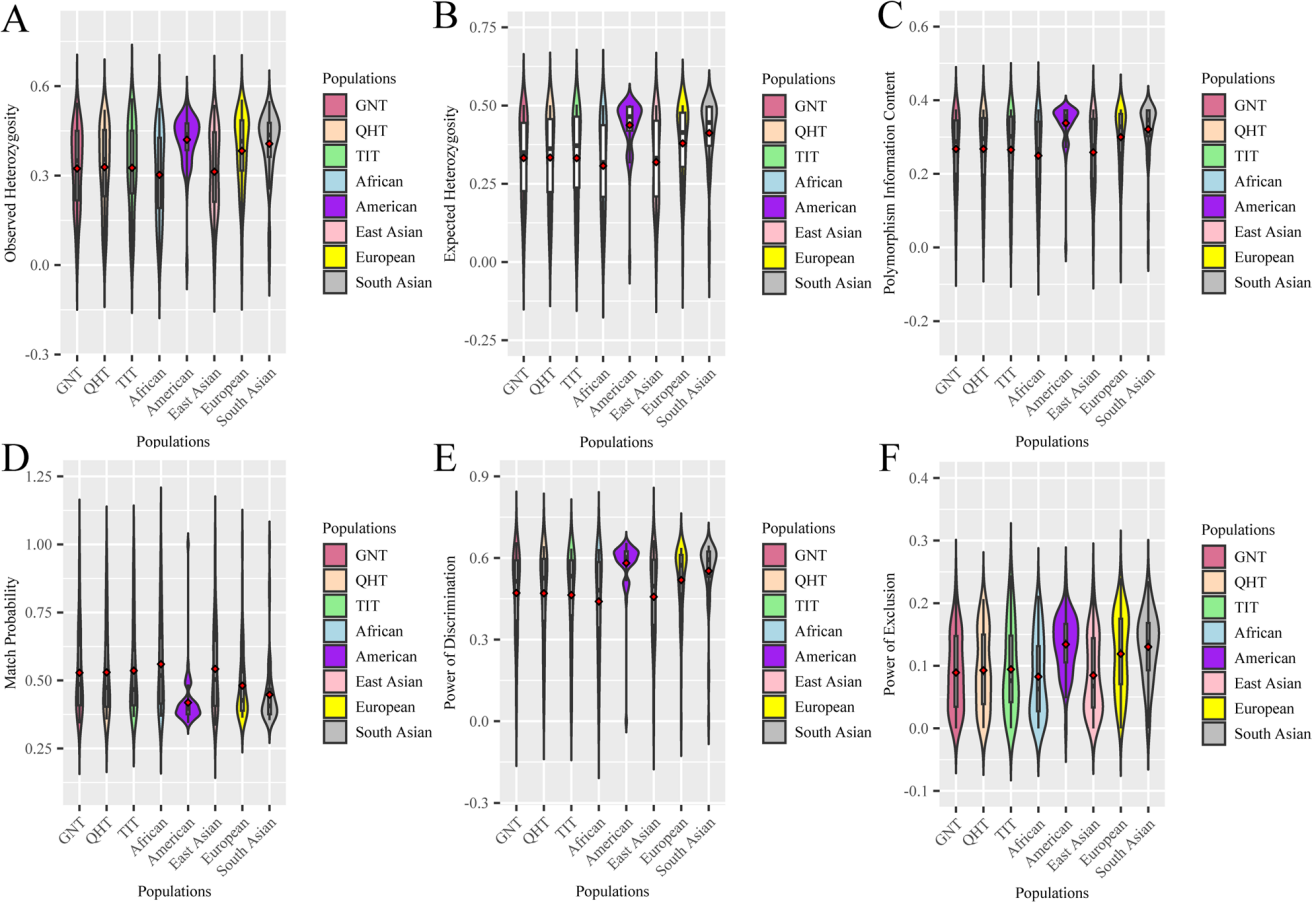
Violin plots of forensic parameters of 56 AIM-InDels in the three Tibetan groups and five intercontinental populations. The distributions of Ho values (**A**), He values (**B**), PIC values (**C**), MP values (**D**), PD values (**E**), and PE values (**F**) in the three Tibetan groups and five intercontinental populations were presented as violin plots

In present study, we calculated the forensic parameters. The higher the values of Ho, He, PIC, PD, and PE, the more efficacy the makers are in forensic applications. As shown in Table S3, in the GNT group, the values of PD and Ho ranged from 0.0220 (rs5896844) to 0.6575 (rs67185986), and 0.0111 (rs5896844) to 0.5444 (rs3842715); in the QHT group, from 0.0540 (rs5896844) to 0.6434 (rs3045215), and 0.0278 (rs5896844) to 0.5208 (rs35794676); in the TIT group, from 0.0384 (rs5896844) to 0.6342 (rs10533245), and 0.0196 (rs5896844) to 0.5588 (rs67185986 and rs5868949), respectively. The overall distributions of forensic parameters are shown in Fig. 3. The findings showed that the distributions of forensic parameters in the three Tibetan groups were generally consistent, which was consistent with those in the East Asian populations. The cumulative power of discrimination (CPD) and cumulative probability of exclusion (CPE) values were calculated for 56 AIM-InDels in the three Tibetan groups. The CPD values were 0.9999999999999999, 0.9999999999999999 and 0.9999999999999999 in the GNT, QHT, and TIT groups, respectively; and the CPE values were 0.995212, 0.996221 and 0.996688, respectively. This suggested that in the three Tibetan groups, the 56 AIM-InDels could be effectively utilized for individual identification, yet lack sufficient efficacy for paternity testing. Detailed forensic parameter information is listed in Table S3.

### Testing of full siblings, half siblings and unrelated individuals based on 56 AIM-InDels

**Fig. 4:**
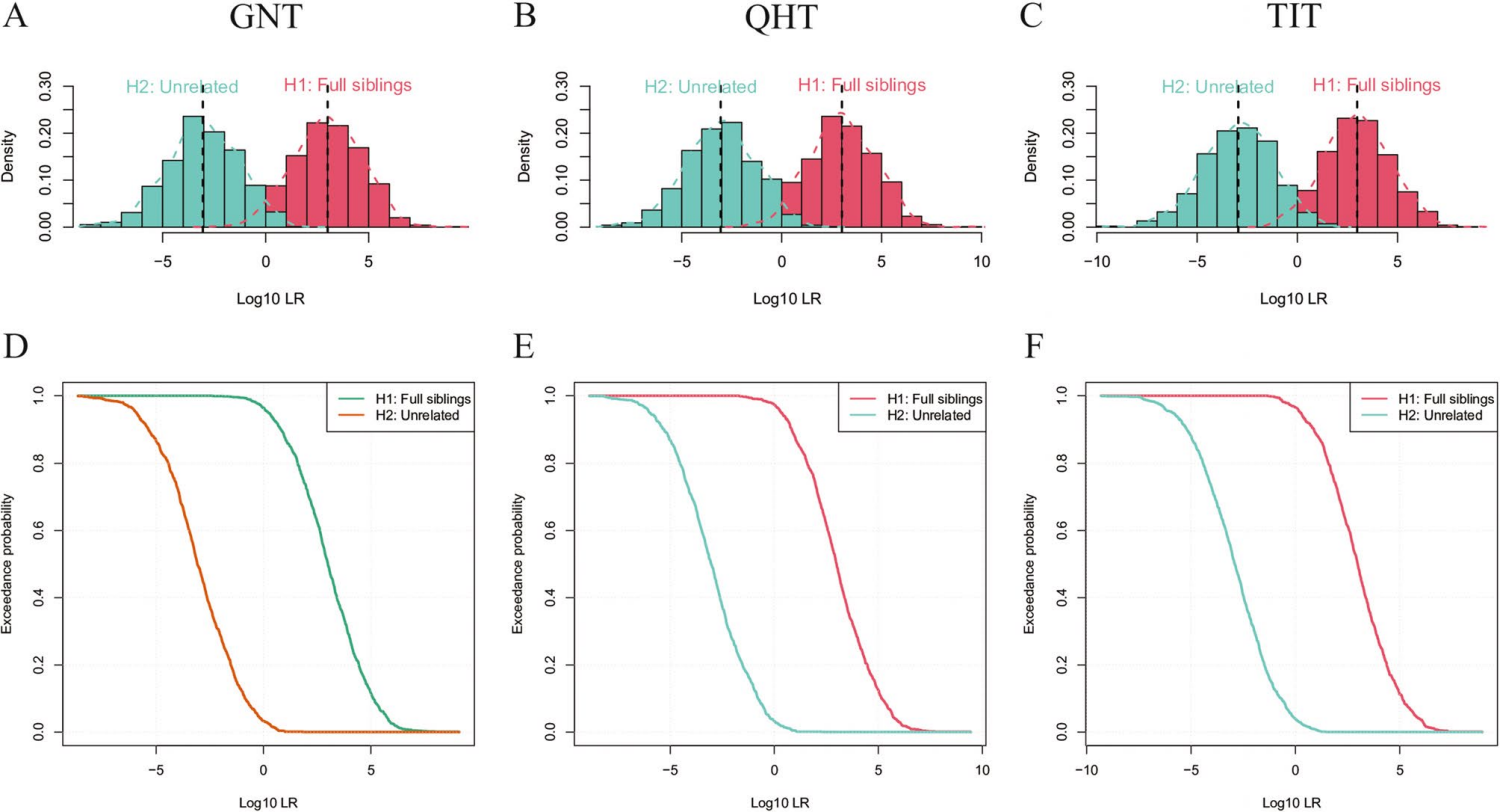
log10 (LR) distribution of 1000 simulated pairs of full siblings and unrelated individuals from three Tibetan groups

We simulated 1000 pairs of full siblings and unrelated individuals, half siblings and unrelated individuals in each of the three Tibetan groups. As shown in Fig. 4, the log (10) LR distributions of full siblings and unrelated individuals partially overlap in all three Tibetan groups. As shown in Table S8, when the LR thresholds are set to 1, 10, 100, 1000 and 10,000, respectively, the accuracy rates for full-sibling identifications were 96.4%, 97.3%, 96.5%; 87.6%, 87.8%, 88.7%; 72.4%, 73.3%, 72.6%; 50.2%, 49.7%, 49.4%; 28.6%, 28.2%, 26.7% in GNT, QHT, and TIT groups, respectively. However, in the identification of half siblings and unrelated individuals, the overlap of log (10) LR distributions is significant in the three Tibetan groups (Fig. S2). As shown in Table S8, when the LR threshold is set to 1, the accuracy rates for half siblings and unrelated individual identifications of GNT, QHT and TIT groups are 80.8%, 84.1%, and 81.5%, respectively.

### The degree of genetic differentiation among the 29 populations and I_n_ values at 56 AIM-InDels

**Fig. 5:**
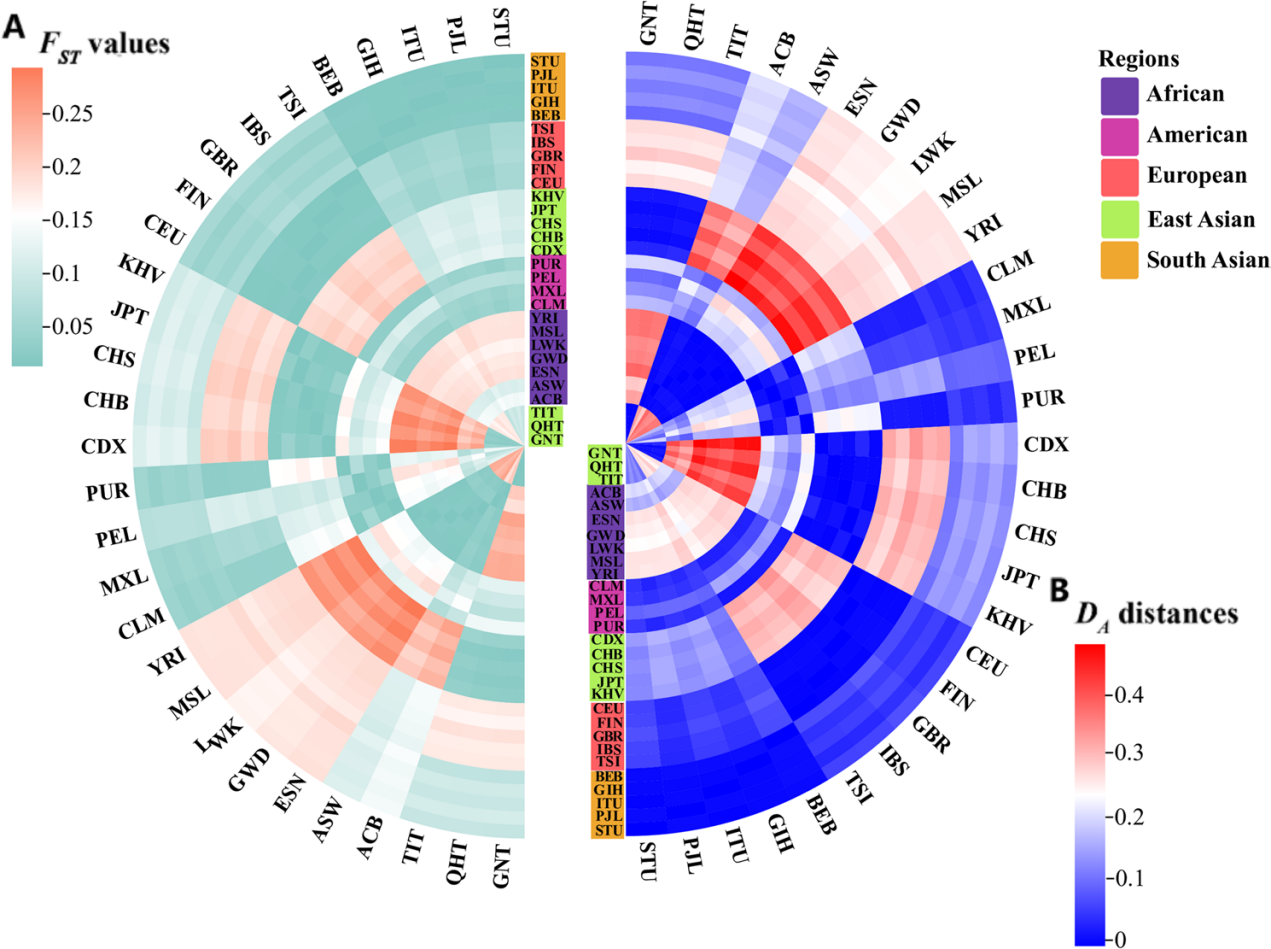
The heatmaps based on the pairwise *F*_ST_ values and *D*_A_ distances among the three Tibetan groups and 26 reference populations. (**A**) Heat map based on the pairwise *F*_*ST*_ values. (**B**) Heat map based on the pairwise *D*_*A*_ distance

*F*_*ST*_ values and *D*_*A*_ distances can measure the degree of genetic differentiation between pairwise populations. The larger *F*_*ST*_ values and *D*_*A*_ distances indicate the greater genetic differences between the two populations, and vice versa. As shown in Fig. 5, the distribution patterns of *F*_*ST*_ values and *D*_*A*_ distances are similar. It could be found that the *F*_*ST*_ values and *D*_*A*_ distances among the same intercontinental populations were smaller, showing that they were less genetically different from each other. Compared to the reference populations, the GNT, QHT and TIT groups were observed the lowest *F*_*ST*_ values and *D*_*A*_ distances with the CHB population (*F*_*ST*_: 0.008, 0.009 and 0.012, respectively; *D*_*A*_ distance: 0.008, 0.008 and 0.011, respectively). The three Tibetan groups were the least genetically different from the five reference East Asian populations compared to the other intercontinental populations. The detailed information about *F*_*ST*_ values and *D*_*A*_ distances is shown in Tables S9 and S10.

**Fig. 6:**
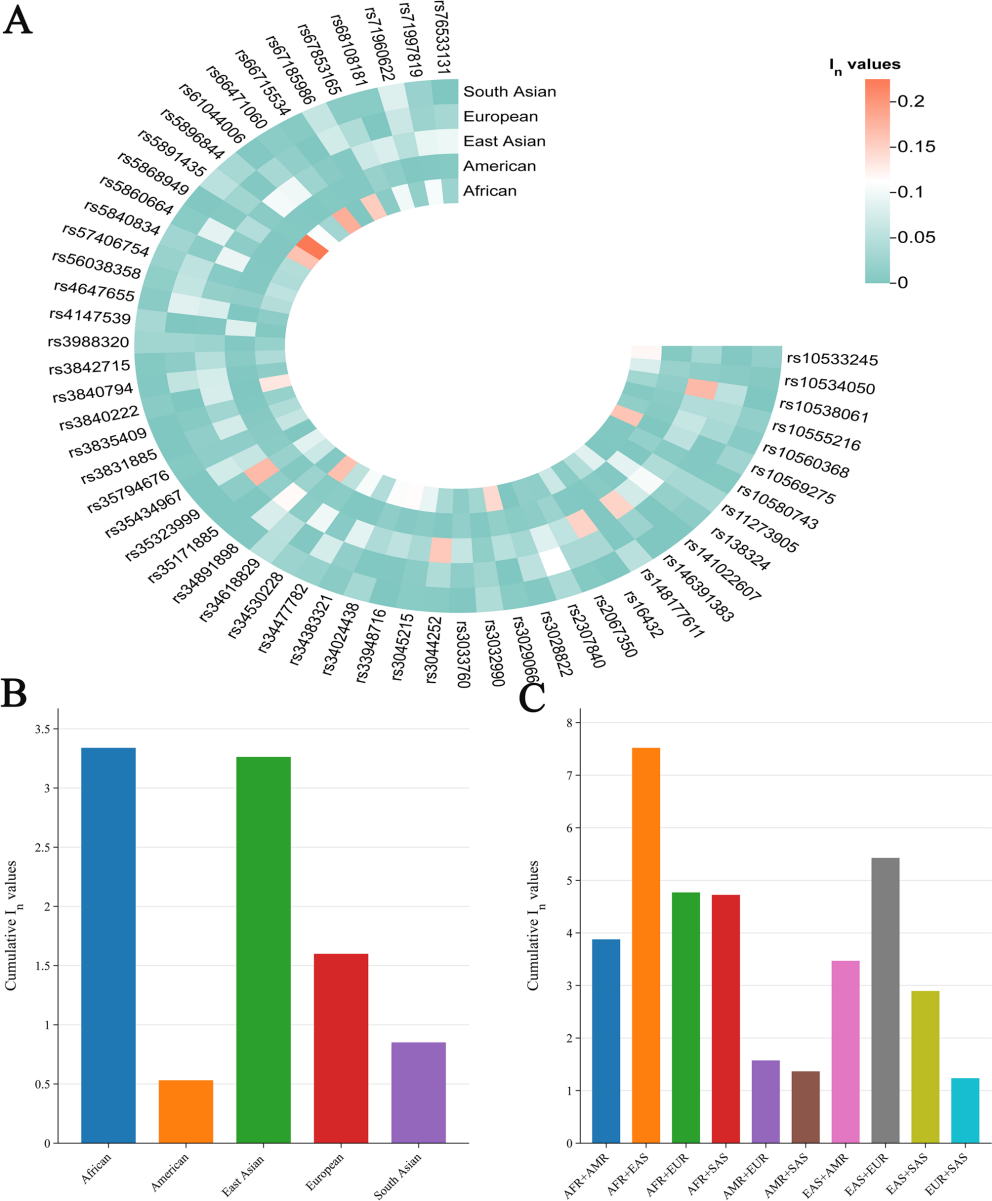
*I*_*n*_ values of the 56 AIM-InDels and cumulative *I*_*n*_ values for the panel. (**A**) *I*_*n*_ values of 56 AIM-InDels in the five intercontinental populations. The color gradient ranged from blue to red, corresponding to *I*_*n*_ values from low to high. (**B**) Cumulative *I*_*n*_ values of 56 AIM-InDels in distinguishing an intercontinental population from other intercontinental populations. (**C**) The cumulative *I*_*n*_ values of the 56 AIM-InDels in distinguishing pairwise intercontinental populations. AFR: African; AMR: American; EAS: East Asian; EUR: European; SAS: South Asian

The *I*_*n*_ value is used to evaluate the efficacy of different loci in distinguishing different populations. A higher *I*_*n*_ value means that the locus is more effective at distinguishing between populations. The *I*_*n*_ value can be converted into the population-specific difference (PSD) value, specifically, the *I*_*n*_ value divided by ln (2) is the PSD value [31]. Detailed *I*_*n*_ values are listed in Table S11. A heatmap of *I*_*n*_ values for the 56 AIM-InDel loci is shown in Fig. 6A. We could note that the African, East Asian, and European populations exhibited higher *I*_*n*_ values at some AIM-InDels. In African populations, for example, *I*_*n*_ values of rs67186986, rs10569275, rs34618829, rs5868049 and rs66471060 loci were greater than 0.15; in East Asian populations, rs3044252, rs35323999 and rs10538061 loci had *I*_*n*_ values greater than 0.15. As shown in Fig. 6B, the cumulative *I*_*n*_ values were greater than 3.0 in African and East Asian populations, demonstrating that the AIM-InDel panel was more effective in distinguishing Africans from non-Africans and East Asians from non-East Asians. In addition, the cumulative *I*_*n*_ value for the European populations was greater than 1.5, suggesting that the AIM-InDel panel can distinguish Europeans to some extent. We also compared population differences among pairwise intercontinental populations. As shown in Fig. 6C, African and East Asian populations had the highest population differences, followed by East Asian and European populations.

### PCA and MDS analysis on basis of 56 AIM-InDels

**Fig. 7:**
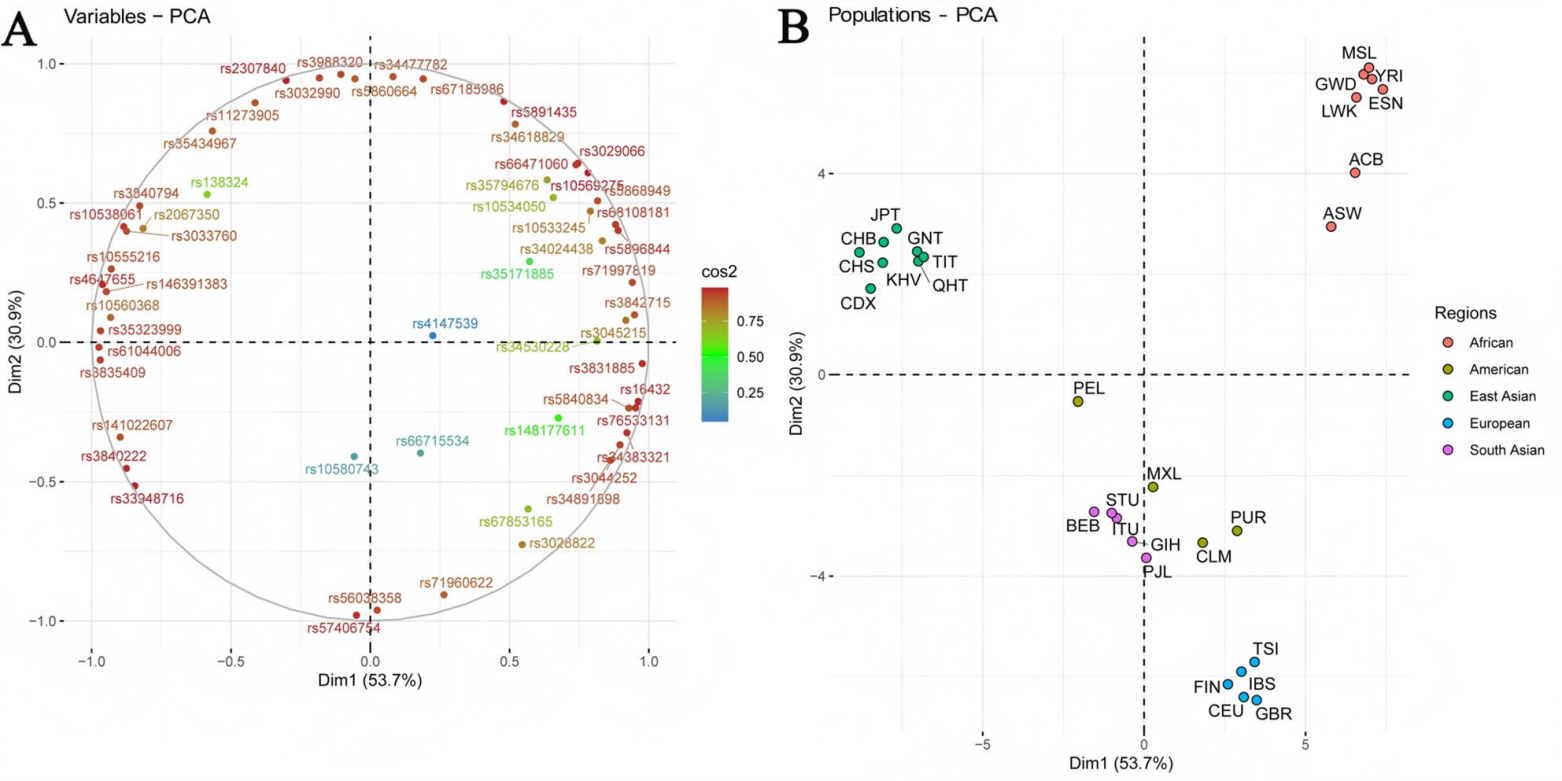
Plot of cos2 values for different variables and population-level PCA plot for the three Tibetan populations and 26 reference populations. **A** Plot of cos2 values for the 56 AIM-InDels. The gradual range in color from blue to red at different points meant that the cos2 values of AIM-InDels were from low to high. **B** Population-level PCA plot of the three Tibetan groups and 26 reference populations. Dots of different sizes represented the contributions of different populations to the principal component

The population-level PCA result is shown in Fig. 7. The larger cos2 value of AIM-InDel was closer to the circle’s border, and the greater its contribution to the principal component (Fig. 7A). We could see that most AIM-InDels were close to the boundary of circle, indicating that they played relatively important roles in the PCA. The East Asian, African and European populations were in the upper left, upper right and lower right of the figure, respectively (Fig. 7B). The three Tibetan groups clustered with the East Asian populations. Furthermore, an individual-level PCA plot was generated using the raw genotype data of the Tibetan and reference individuals. As shown in Fig. S3A, individuals from East Asian and African populations cluster separately, while individuals from the remaining three intercontinental populations could not be well distinguished. Based on pairwise *F*_*ST*_ values, the MDS analysis of 29 populations was also performed. As shown in Fig. S3B, the clustering result is similar to the population-level PCA plot.

### Reconstruction of the NJ tree and inference of gene flow events

**Fig. 8:**
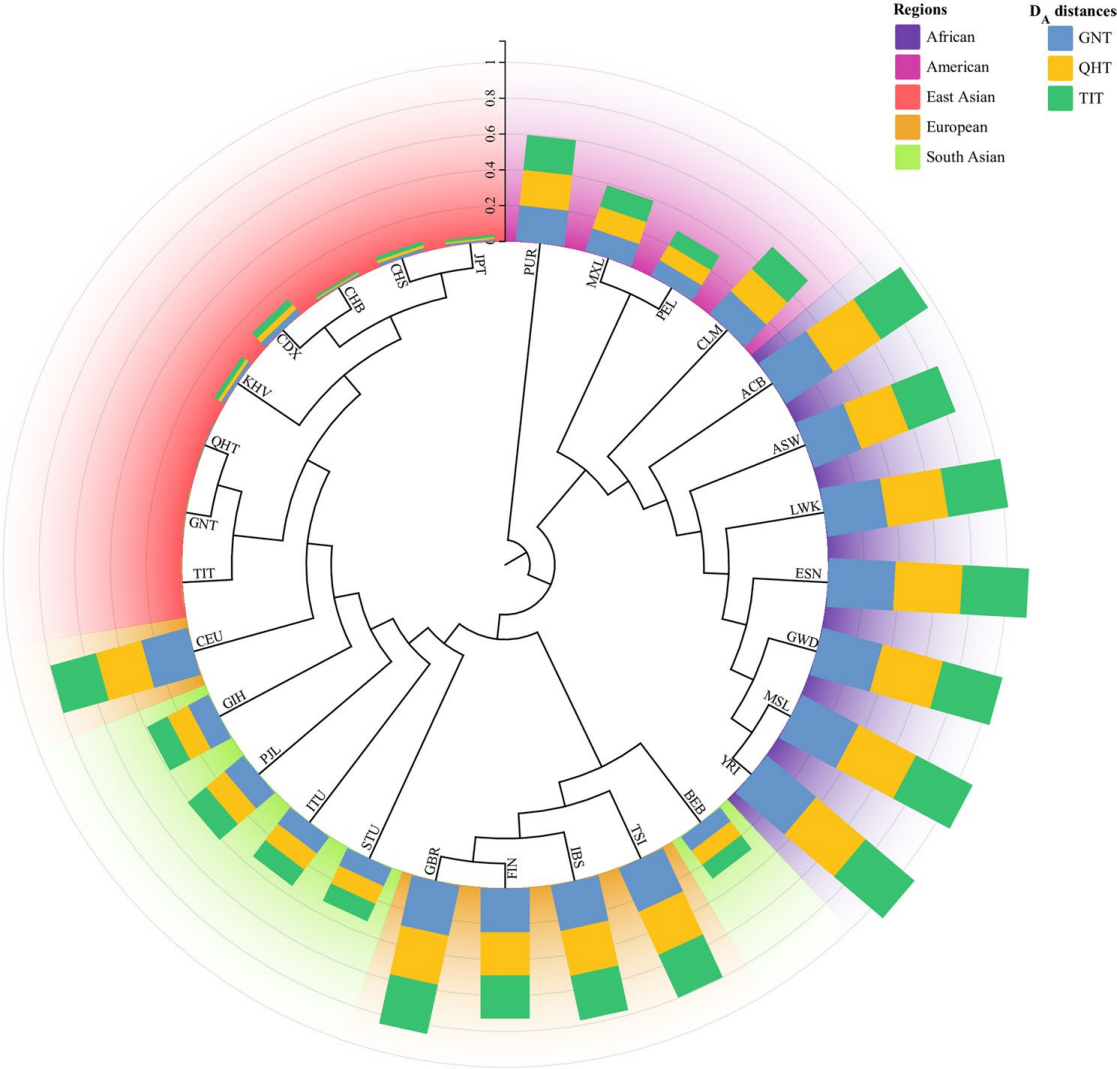
Neighbor-joining tree reconstructed from three Tibetan groups and 26 reference populations. A rooted neighbor-joining tree based on pairwise *D*_*A*_ distances of the three Tibetan groups and 26 reference populations. The stacked bar plot in the outer circle showed the *D*_*A*_ distances among the 29 populations and the GNT, QHT, and TIT groups, respectively

As shown in Fig. 8, the NJ tree shows that different intercontinental populations could be clustered together separately. The three Tibetan groups firstly clustered with five East Asian reference populations, and then with other intercontinental populations. The stacked bar plot of *D*_*A*_ distances showed that the three Tibetan groups had the smallest genetic distances from the East Asian populations, and the largest genetic distances from the African populations. For the gene flow event analyses by Treemix software, the optimal number of gene flow events for the 29 populations is determined to be two (Fig. S4A). We observed that the three Tibetan groups and five reference populations (CHB, CHS, CDX, JPT and KHV populations) were located in the same sub-branch, indicating that their genetic structures were more similar (Figs. S4C and S4D). The residual heatmap indicates the discrepancy between the fitted model and actual situation, with a residual close to 0, indicating a more realistic situation. In Figs. S4E and S4F, most of the residuals of the maximum likelihood (ML) trees were located near 0, indicating that this ML tree was consistent with the actual situation.

### Population genetic structure analysis

**Fig. 9:**
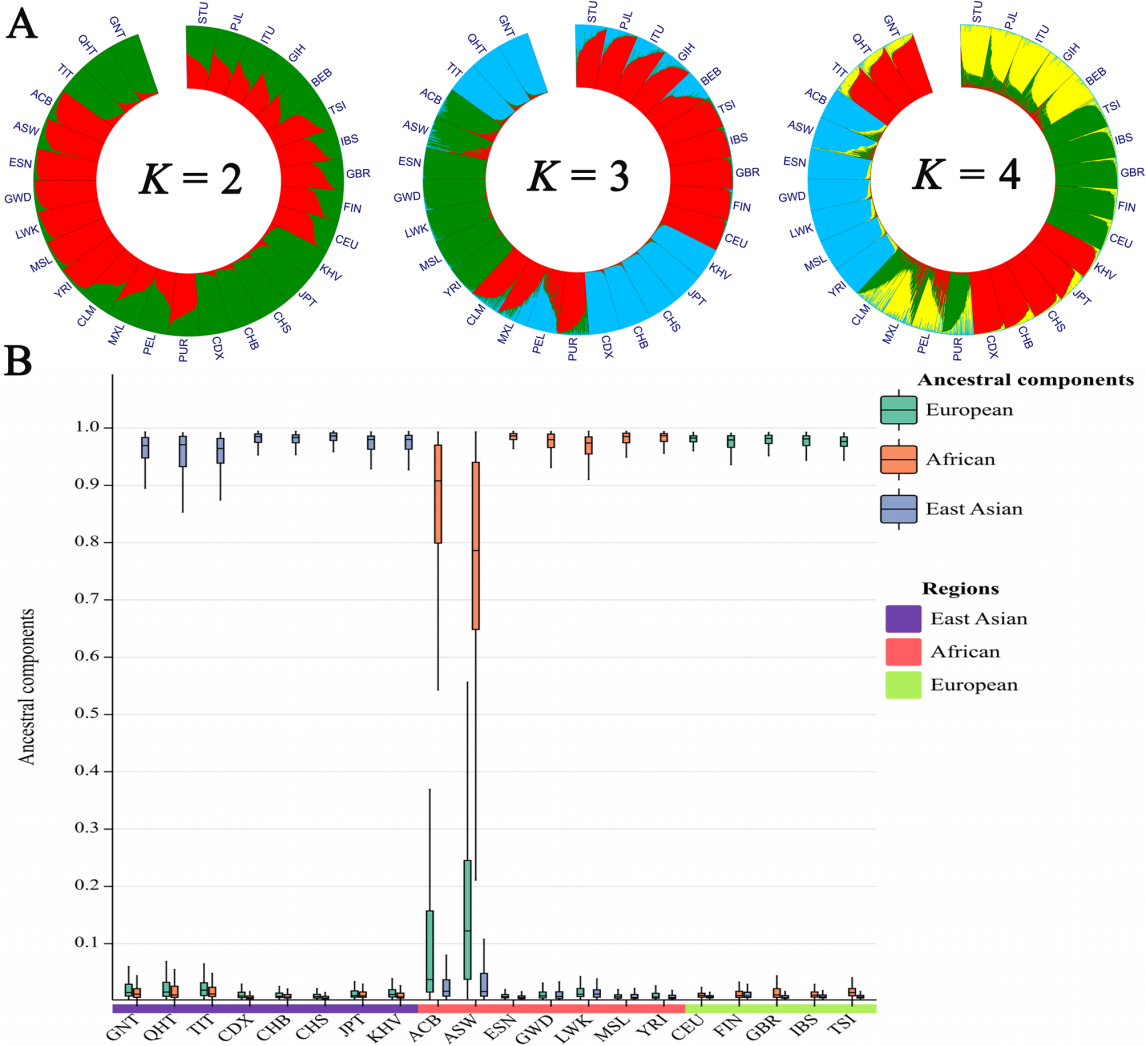
STRUCTURE analyses of different *K* values for the three Tibetan groups and 26 reference populations. (**A**) When *K* = 2, 3, 4, STRUCTURE analyses were performed based on the raw genotype data of 56 AIM-InDels for the three Tibetan groups and 26 reference populations. (**B**) When *K* = 3, the ancestral components of the African, East Asian, and European populations were shown in the box plot

**Fig. 10:**
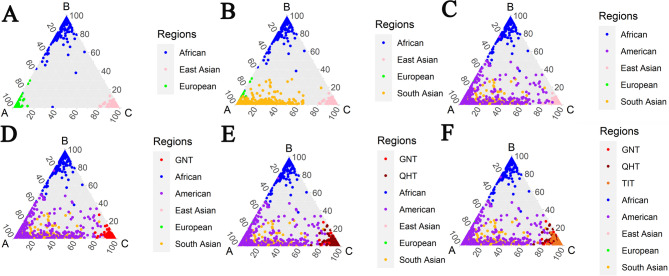
Clustering analyses of ternary plots of different populations when *K* = 3. (**A**) Ternary plot of ancestral components of African, East Asian, and European populations. (**B**) Ternary plot of ancestral components of African, East Asian, European, and South Asian populations. (**C**) Ternary plot of ancestral components of African, American, East Asian, European, and South Asian populations. (**D**) Ternary plot of ancestral components of African, American, East Asian, European, South Asian populations, and GNT group. (**E**) Ternary plot of ancestral components of African, American, East Asian, European, South Asian populations, and GNT, QHT groups. (**F**) Ternary plot of ancestral components of African, American, East Asian, European, South Asian populations, and GNT, QHT, TIT groups

We used the STRUCTURE program to further investigate the genetic structures of the three Tibetan groups. According to the InP*K* and delta*K* plots (Fig. S5), the optimal *K* value is three. As shown in Fig. 9A, when *K* = 3, the African, European, and East Asian populations could be well distinguished, while the American and South Asian populations displayed mixed ancestral components. It was worth noting that, regardless of the *K* values, the American populations remained in patterns of mixed ancestral components (Fig. S6). We further analyzed the ancestral components of African, East Asian, and European populations when *K* = 3. Except for two African populations (ACB and ASW populations), all other populations show almost a single ancestral component. (Fig. 9B). In addition, a series of ternary plots was used to further dissect the clustering patterns of individuals from different continents when *K* = 3. As shown in Fig. 10, individuals from the three Tibetan groups overlap with East Asian individuals.

## Discussion

In forensic practice, it is important to perform BGA inference on unknown samples. In this study, we used a 56 AIM-InDel panel to explore the efficacy of BGA inference and to assess the genetic structures of GNT, QHT, and TIT groups. First, we used the SVM model to evaluate the BGA inference efficacy of the 56 AIM-InDels. In the absense of noisy labels, the 56 AIM-InDels performed best in classifying African and East Asian individuals, followed by European and South Asian individuals. When noisy labels were added, with the percentage of noisy labels increasing, the performance of the model would decrease accordingly. We observed that when the percentage of noisy labels was less than 50%, the SVM model still had good robustness. There were two possible reasons for this result. The first aspect​​ was the implementation of the Radial Basis Function (RBF) kernel in our SVM model. Its characteristic is that only training data points in close proximity to a test sample exert significant influence on its label, thereby conferring robustness [42]. The second aspect​ was that we observed that when the labels were shuffled, some noisy data still had the same labels as the original data (for example, in 50% of cases, the true percentage of noisy labels was about 35%). In summary, among all five intercontinental populations, the 56 AIM-InDels performed best for the classification of African and East Asian individuals, followed by European and South Asian individuals. Studies in three Tibetan groups also demonstrated that the panel could be applied to forensic practice for BGA inference.

Then, we calculated the CPD and CPE values of 56 AIM-InDels. A forensic panel is considered applicable for individual identification and paternity testing only if its CDP value exceeds 0.9999 and CPE value surpasses 0.9999. Therefore, in our study, the 56 AIM-InDels could be effectively used for individual identification in the three Tibetan groups; however, they had inadequate power for parentage testing. Furthermore, based on the allele frequency data of 56 AIM-InDels, the identification abilities between full siblings and unrelated individuals, as well as between half-siblings and unrelated individuals, were respectively simulated. The present results showed that this panel could be used as an important tool for the identification of full sibling and unrelated individuals, but it has low efficiency for the identification of half sibling and unrelated individuals.

Next, we used several methods to reveal the genetic backgrounds of the GNT, QHT, and TIT groups. Insertion allele frequency distributions revealed that the three Tibetan groups clustered together with the reference East Asian populations **(**Fig. 2**)**. The *I*_*n*_ value is used to assess the efficacy of AIM loci in distinguishing different populations. The results of *I*_*n*_ value showed that 56 AIM- InDels were the most effective in distinguishing African and East Asian populations, followed by European and South Asian populations. The results of *F*_*ST*_ value and *D*_*A*_ distance showed that the three Tibetan groups had the closest genetic distances to the reference East Asian populations. The three Tibetan groups also clustered with the East Asian populations in the PCA, MDS and NJ tree analyses. Further, the three Tibetan groups were genetically closer to each other compared with other East Asian populations. In addition, the results of PCA and MDS indicated that African, East Asian and European populations could be clustered together separately. In PCA, we found several loci with cos2 values greater than 0.9, including rs34477782, rs4647655, and rs35323999, indicating these loci were more promising for BGA inference. The results of STRUCTURE analysis indicated that the three Tibetan groups had similar ancestral compositions to the East Asian populations, whereas the African, East Asian and European populations displayed relatively single ancestral composition, respectively.

Although existing panels can characterize the genetic backgrounds of Tibetan groups, they have some limitations. Liu et al. and Jian et al. utilized the Qiagen Investigator DIPplex kit and a 35 AIM-InDel panel to investigate the genetic structures of the QHT and TIT groups, respectively [19, 20]. In addition, some scholars have used Ion AmpliSeq MH-74 Plex [43] and 39-AIM-InDel panel [44] to achieve BGA inference. In comparison, our panel has the following advantages: (1) the more efficient AIM-InDels are selected to improve the accuracy of BGA inference; (2) all AIM-InDel lengths are less than 200 bp, which facilitate the detection of degraded sample; (3) the panel based on InDel is compatible with the CE platform, which is conducive to its promotion and application. In conclusion, we demonstrated that the 56 AIM-InDel panel could fulfill the requirements for forensic individual identification as well as BGA inference and revealed that the genetic structures of GNT, QHT and TIT groups were similar to those of East Asian populations.

## Conclusions

In conclusion, we explored the BGA inference performance of a multiplex PCR panel consisting of 56 autosomal AIM-InDels, three Y-InDels, and one Amelogenin gene, and characterized the genetic structures of GNT, QHT, and TIT groups. The results indicated that the 56 AIM-InDel panel had better performance for BGA inference in African and East Asian individuals. In addition, the research on noisy labels found that the SVM model also has better robustness on African and East Asian populations. The results of population genetic analysis demonstrated that the three Tibetan groups had more similar genetic structures to the reference East Asian populations. The Tibetan data we obtained, especially the Gannan Tibetan data, are of great importance for the research of Tibetan groups in different regions. In the future, the performance of BGA inference in forensic practice needs to be further investigated.

## Supplementary Information

Supplementary Material 1.

Supplementary Material 2.

Supplementary Material 3.

Supplementary Material 4.

Supplementary Material 5.

Supplementary Material 6.

Supplementary Material 7.

Supplementary Material 8.

## Data Availability

The data which support the findings of this research are available from the corresponding author upon reasonable request. The raw genotyping data of individuals are not publicly available due to ethical restrictions.
