# Supplementary figures and images for: Performance of a self-developed panel for biogeographic ancestry inference and dissection of the genetic background of three Tibetan groups
Source: Hereditas. 2025 Dec 5;163:9. doi: 10.1186/s41065-025-00604-3 (PMC12797779; doi:10.1186/s41065-025-00604-3)

A

Individuals – PCA

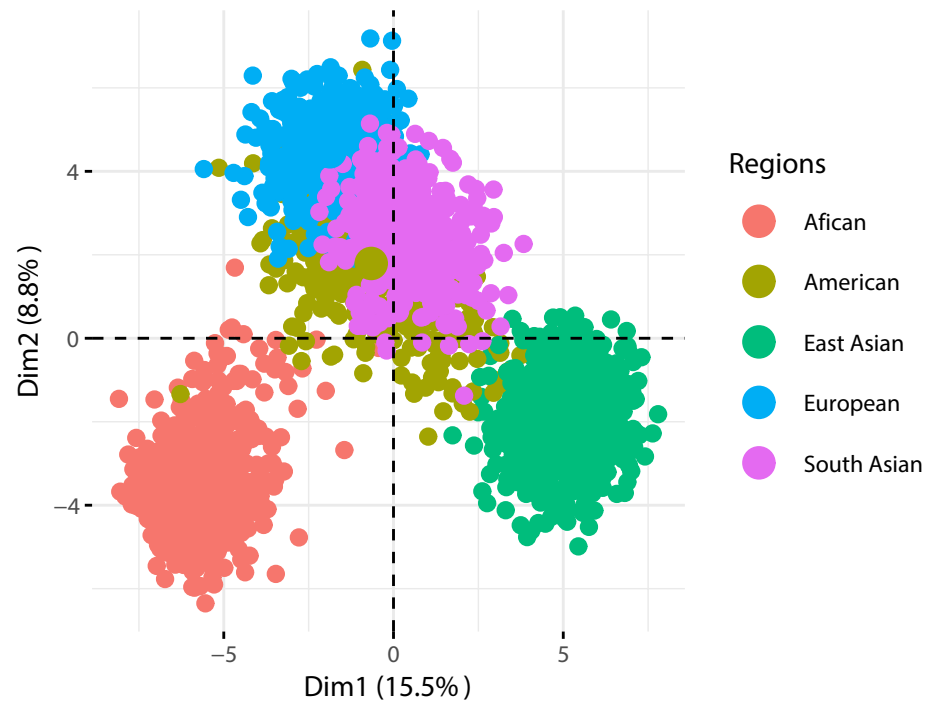

B

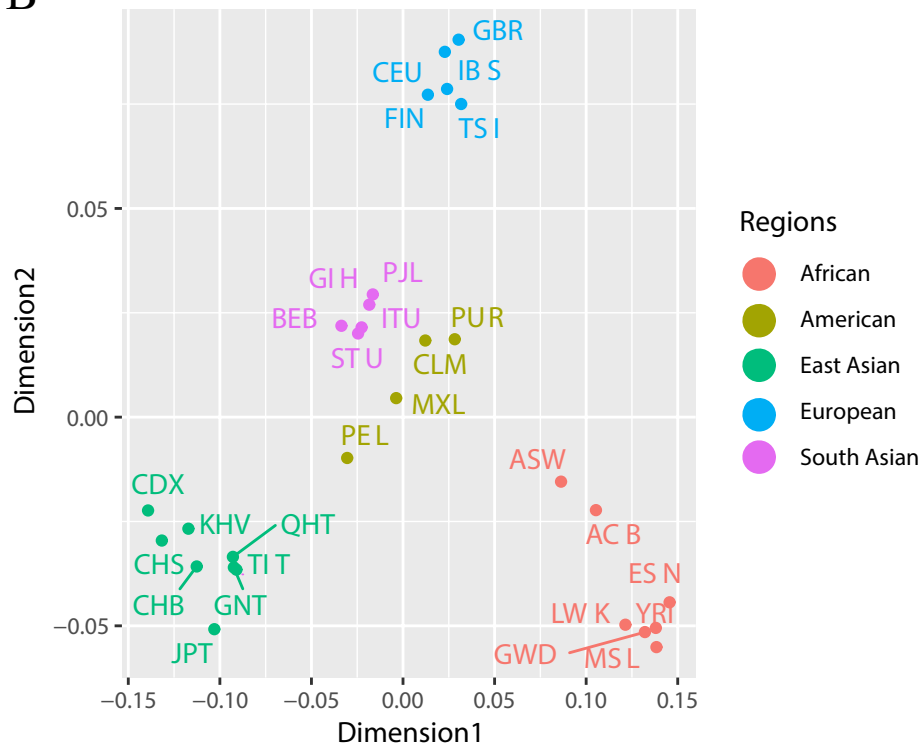

Supplement: Supplementary file 1 — Supplementary Material 1. [file 41065_2025_604_MOESM1_ESM.pdf]

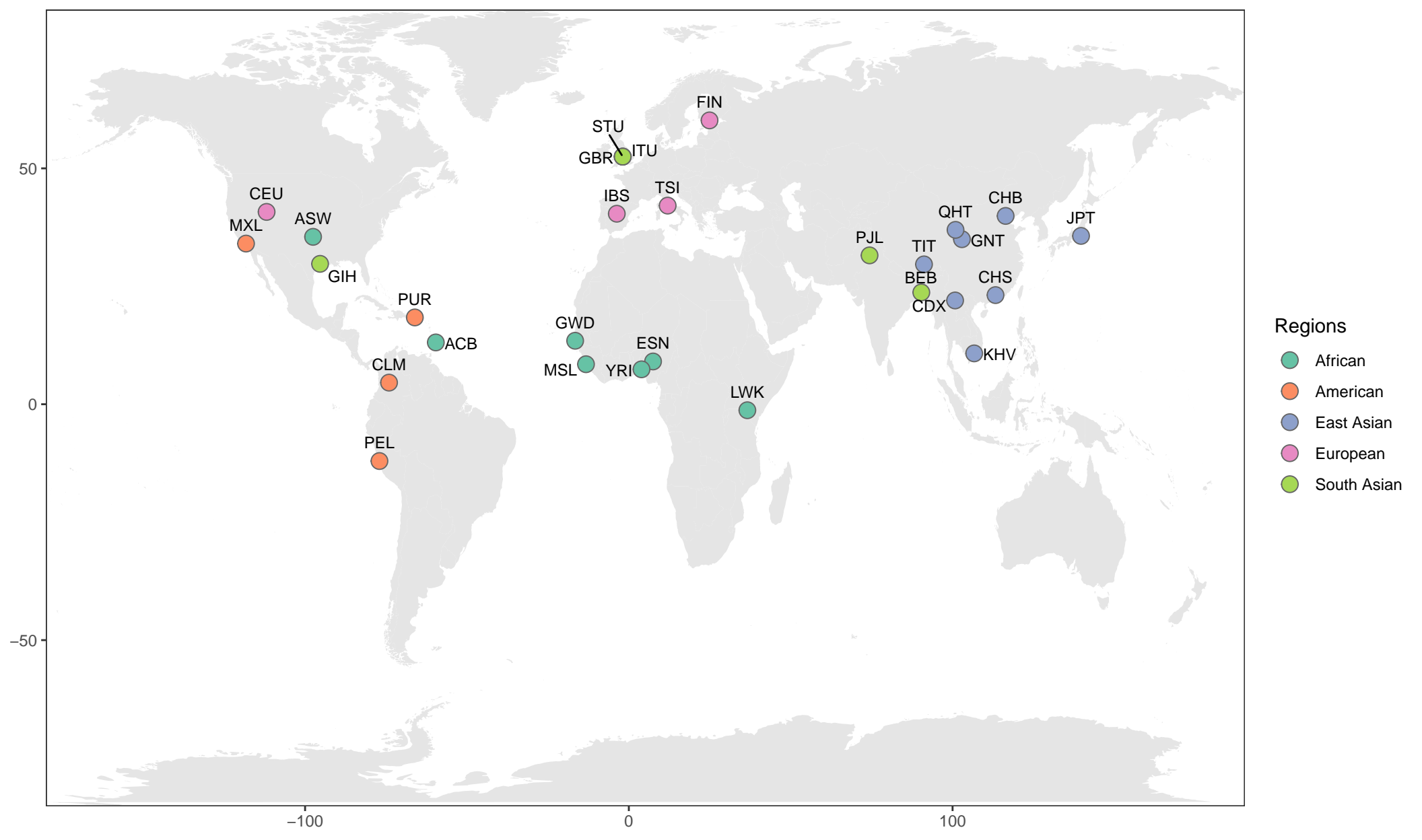

Supplement: Supplementary file 2 — Supplementary Material 2. [file 41065_2025_604_MOESM2_ESM.pdf]

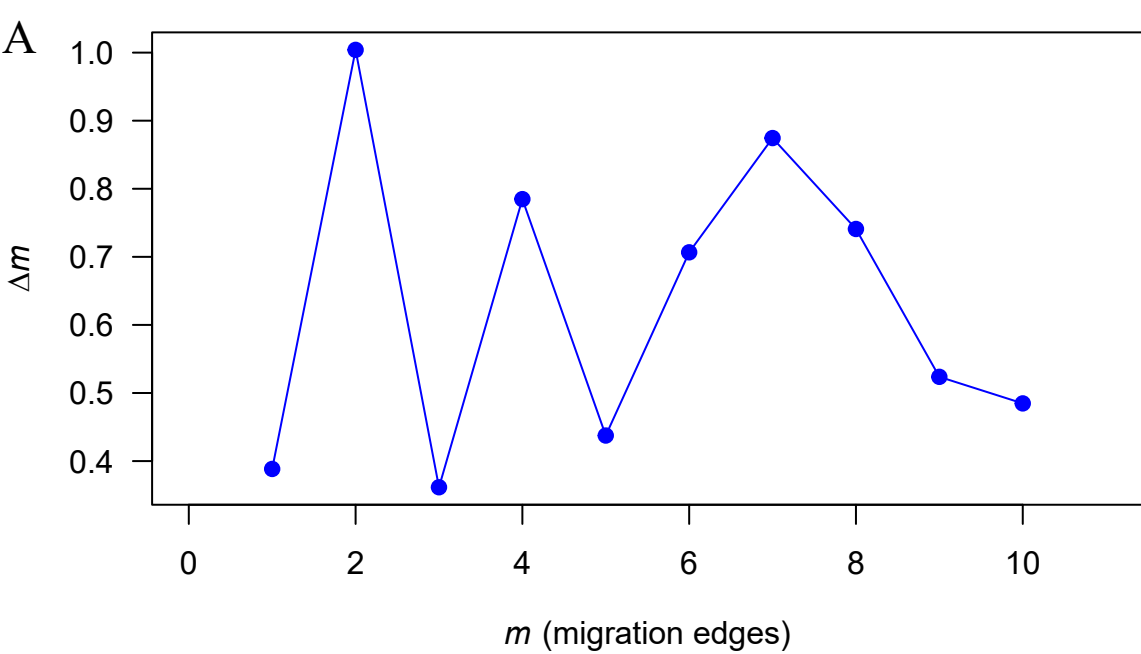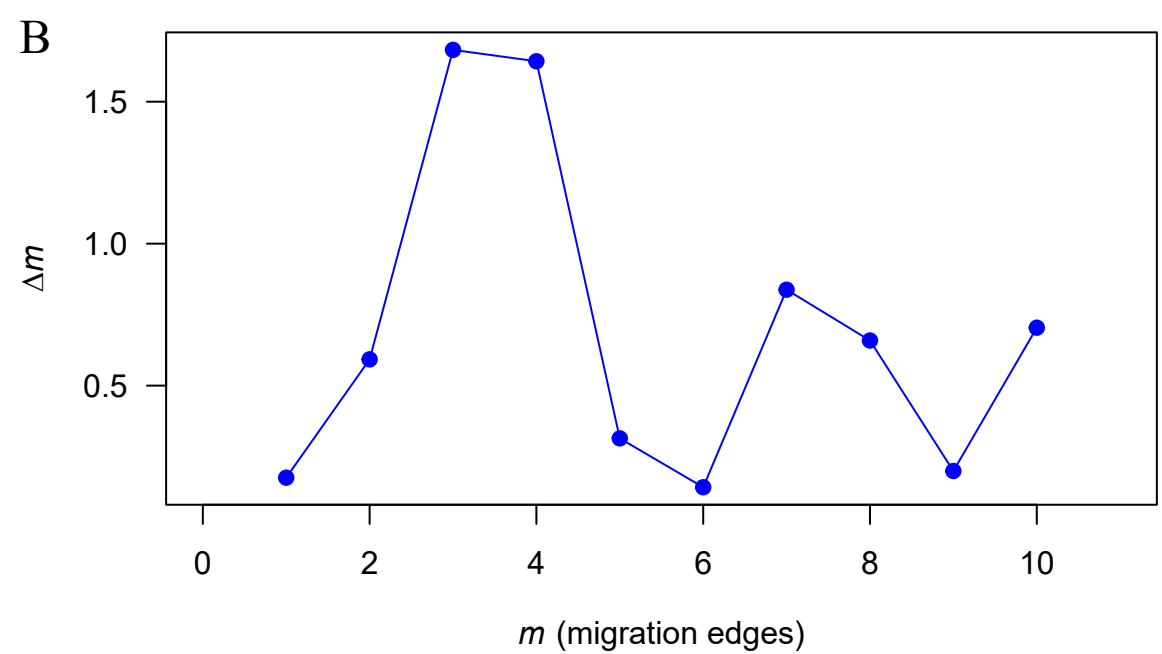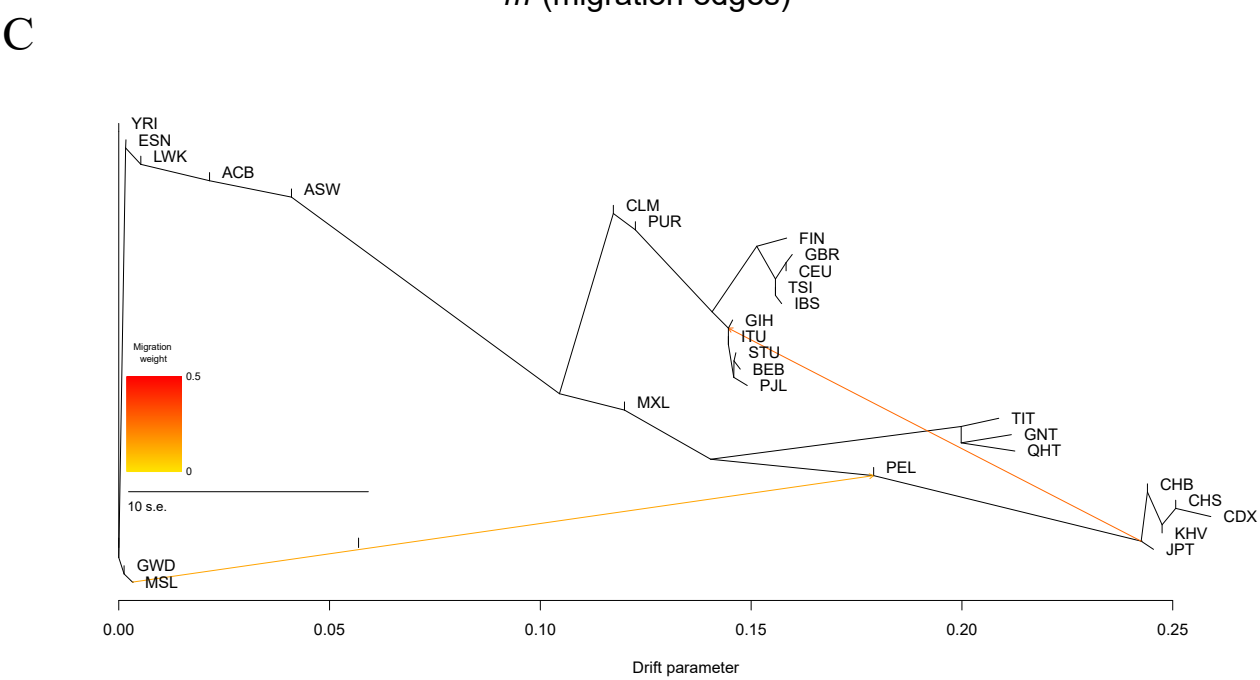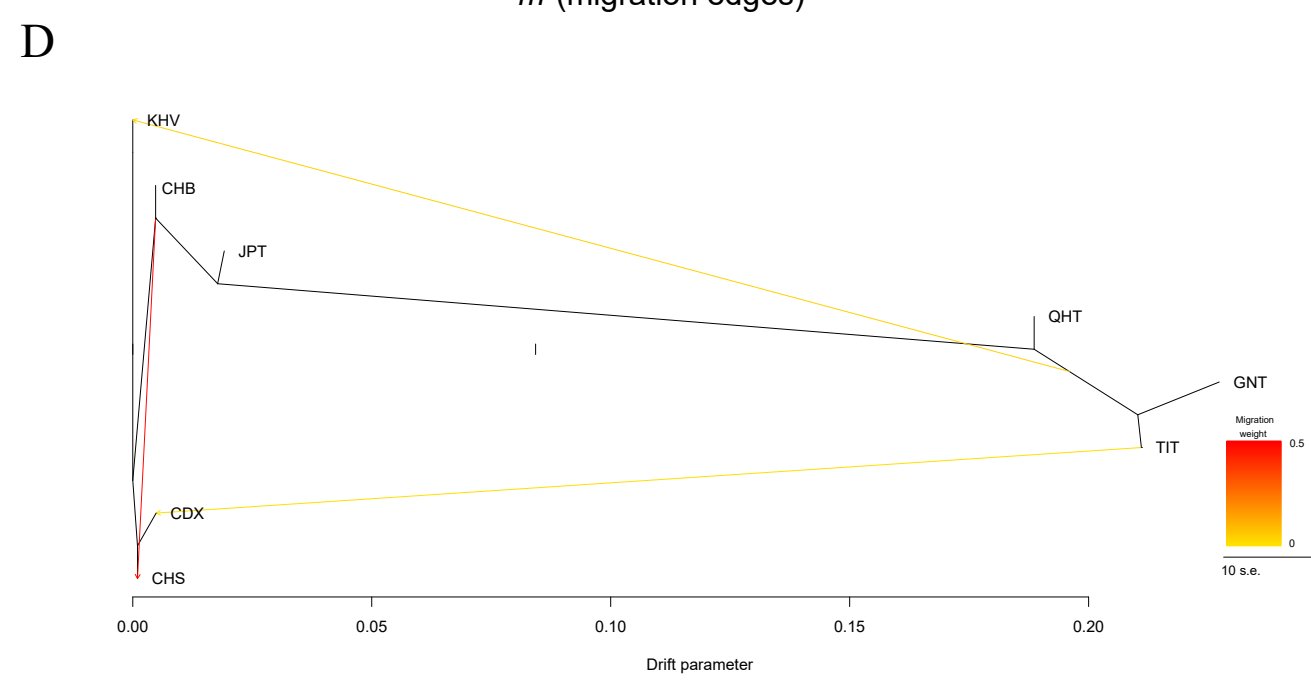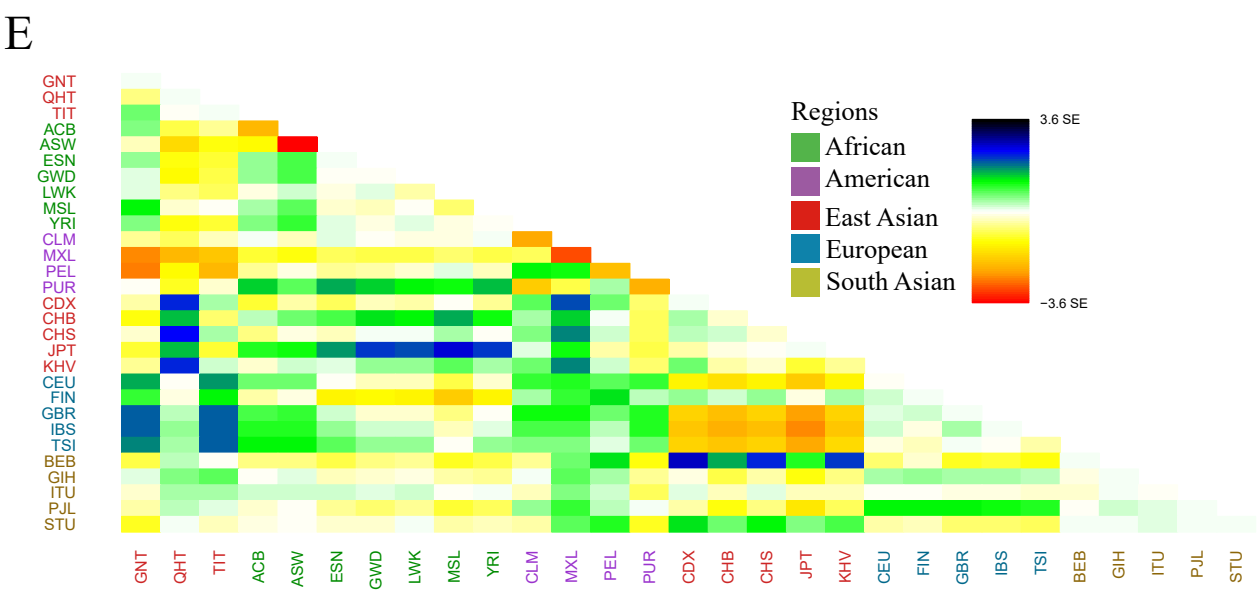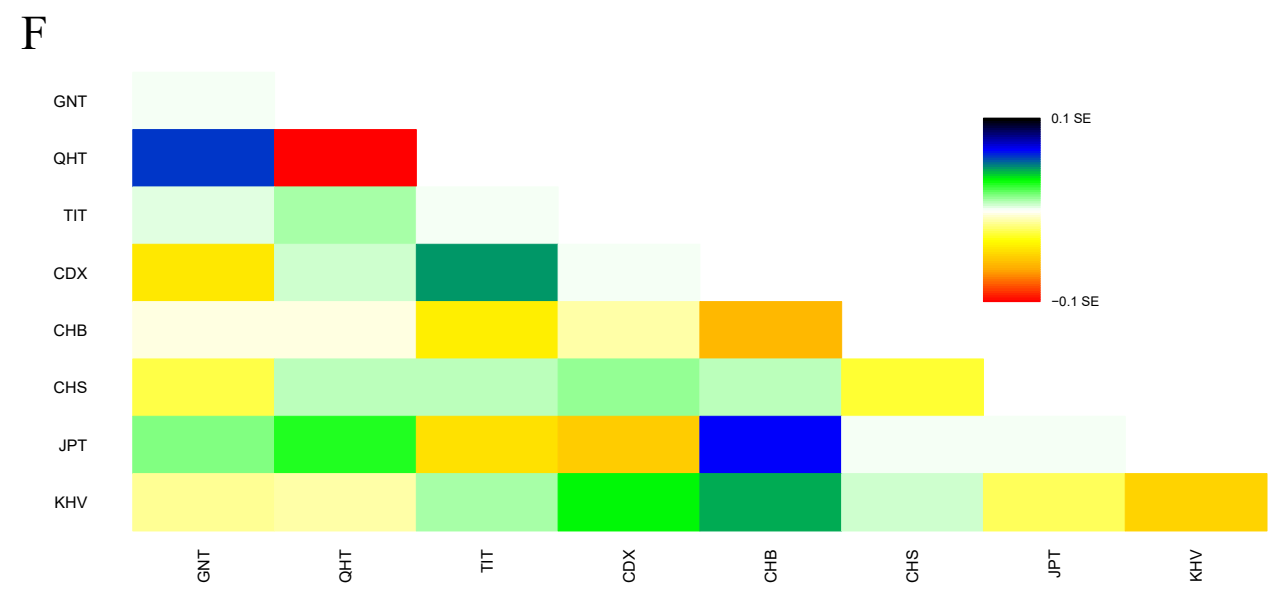

Supplement: Supplementary file 3 — Supplementary Material 3. [file 41065_2025_604_MOESM3_ESM.pdf]

A

GNT

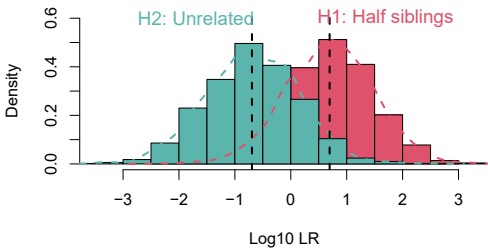

B

QHT

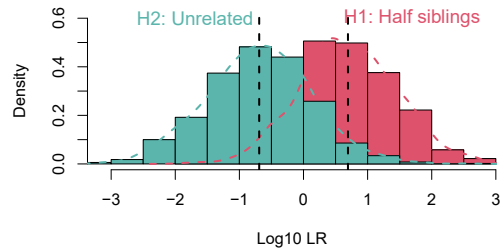

C

TIT

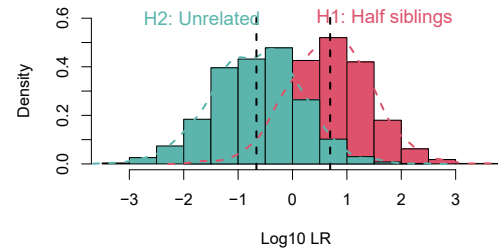

D

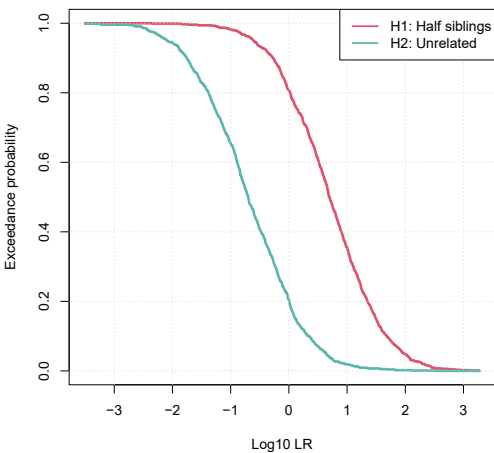

E

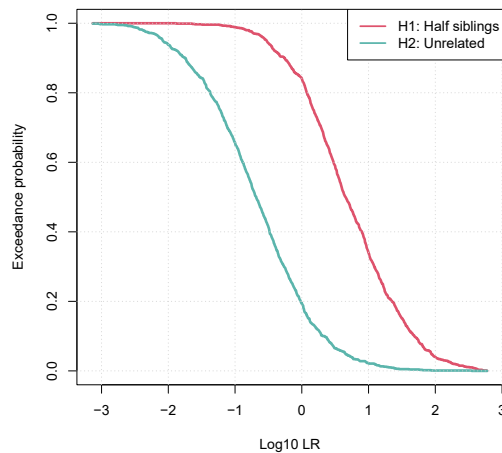

F

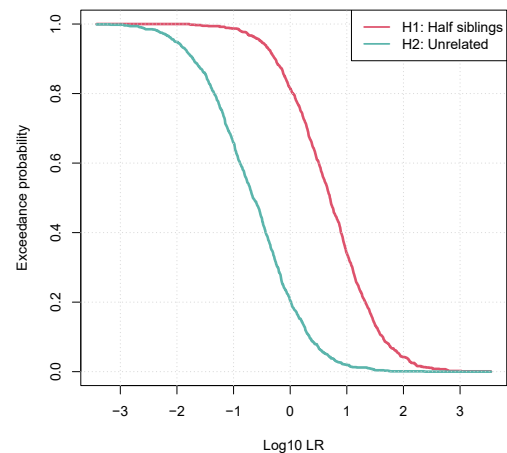

Supplement: Supplementary file 4 — Supplementary Material 4. [file 41065_2025_604_MOESM4_ESM.pdf]

A

Rate of change of the likelihood distribution (mean)

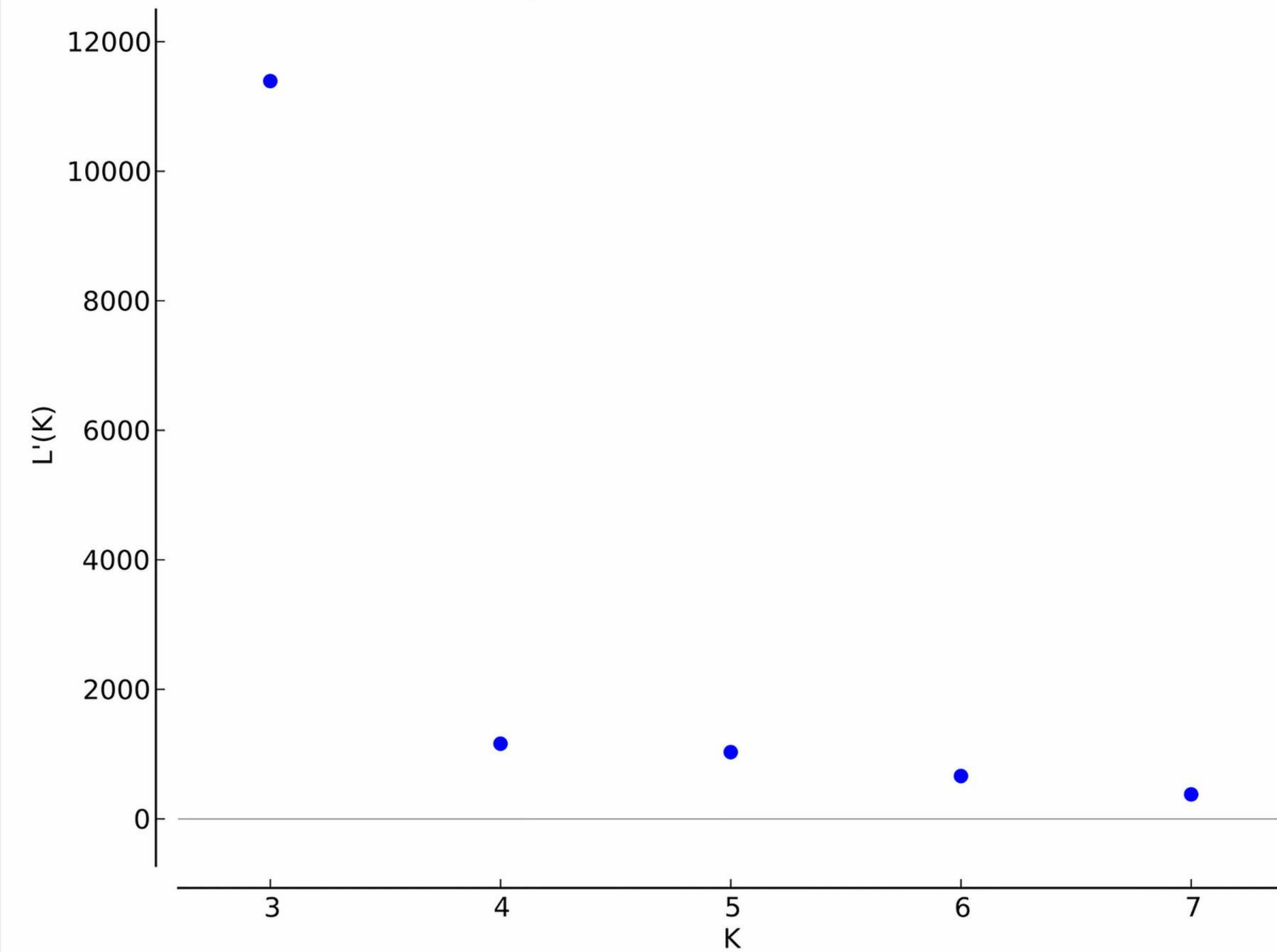

B

DeltaK = mean( $|L''(K)|$ ) / sd(L(K))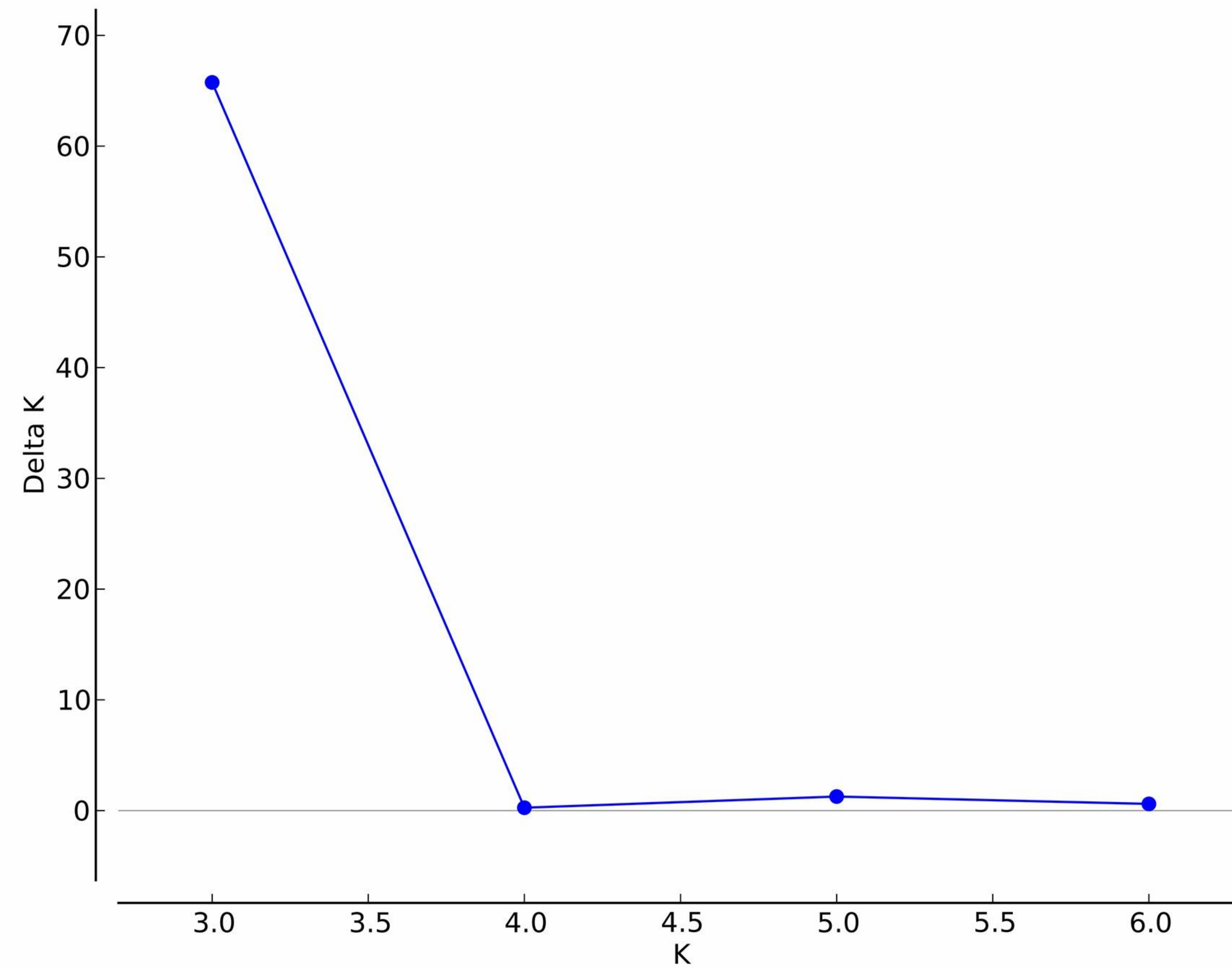

Supplement: Supplementary file 5 — Supplementary Material 5. [file 41065_2025_604_MOESM5_ESM.pdf]

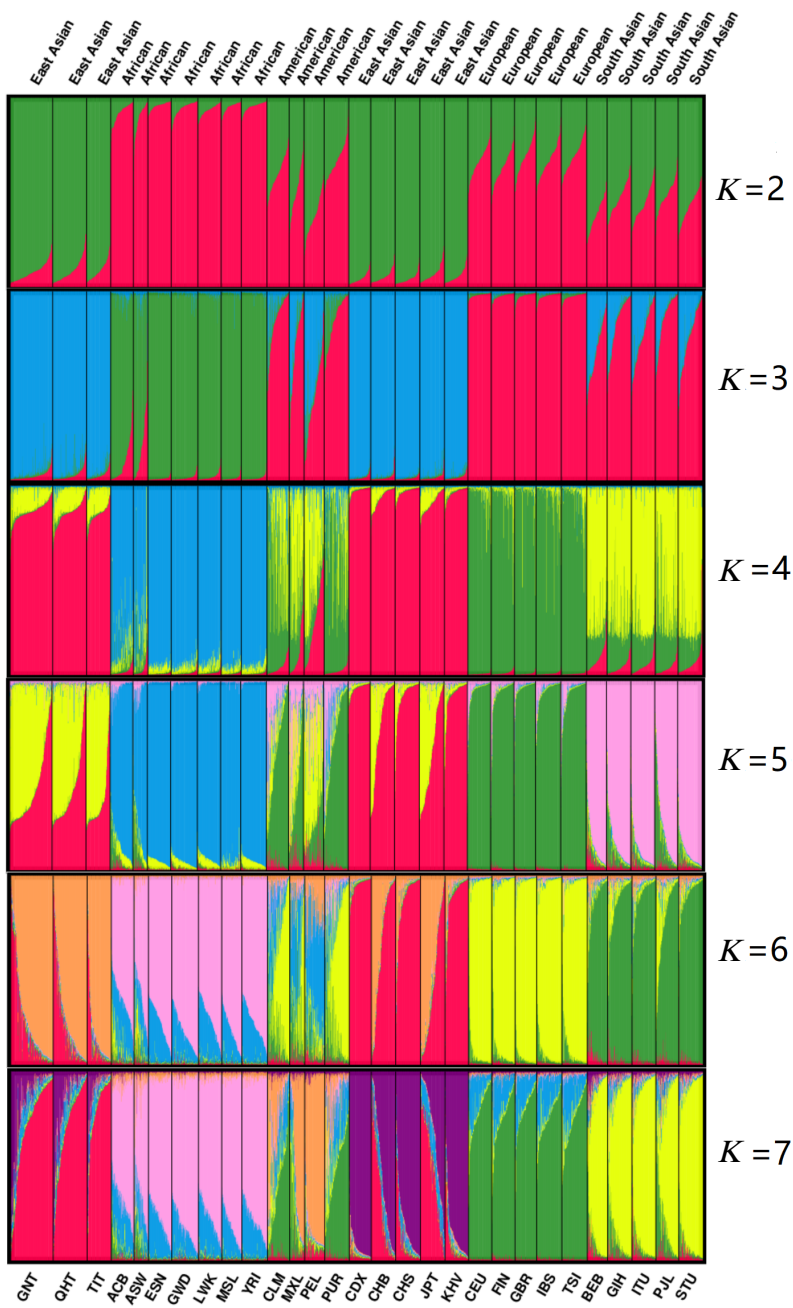

Supplement: Supplementary file 6 — Supplementary Material 6. [file 41065_2025_604_MOESM6_ESM.pdf]
