# Supplementary material for: Performance of a self-developed panel for biogeographic ancestry inference and dissection of the genetic background of three Tibetan groups
Source: Hereditas. 2025 Dec 5;163:9. doi: 10.1186/s41065-025-00604-3 (PMC12797779; doi:10.1186/s41065-025-00604-3)
Supplement: Supplementary file 7 — Supplementary Material 7. [file 41065_2025_604_MOESM7_ESM.docx]

**Figures. S1-6**

**Fig. S1** Map of the geographical locations of all populations.

**Fig. S2** log10 (LR) distribution of 1000 simulated pairs of half siblings and unrelated individuals from three studied groups.

**Fig. S3** Results of PCA at individual-level and MDS at population-level in the three studied groups and 26 reference populations. **(A)** PCA result at the individual-level. **(B)** MDS result at the population-level.

**Fig. S4** Respective Treemix analyses of all 29 populations and eight East Asian populations. The deltaM plots **(A, B)** indicate the optimum numbers of gene flow events for all 29 populations (m = 2) and eight East Asian populations (m = 3). The corresponding maximum likelihood trees were reconstructed when m = 2 **(C)** and m = 3 **(D)**, with each arrow indicating a certain gene flow event and its weight. The residual covariance matrices **(C, D)** were applied to measure the fitting of trees.

**Fig. S5** LnP*K* and Delta*K* values of each *K* based on the 56 AIM-InDels. Structure Harvester program suggested that the optimum *K* value was three.

**Fig. S6** STRUCTURE analysis of the three studied groups and 26 reference populations based on the raw genotype data of 56 AIM-InDels (*K* = 2-7).
